# Fast Spatiotemporal Smoothing of Calcium Measurements in Dendritic Trees

**DOI:** 10.1371/journal.pcbi.1002569

**Published:** 2012-06-28

**Authors:** Eftychios A. Pnevmatikakis, Keith Kelleher, Rebecca Chen, Petter Saggau, Krešimir Josić, Liam Paninski

**Affiliations:** 1 Department of Statistics and Center for Theoretical Neuroscience, Columbia University, New York, New York, United States of America; 2 Department of Mathematics, University of Houston, Houston, Texas, United States of America; 3 Department of Neuroscience, Baylor College of Medicine, Houston, Texas, United States of America; Indiana University, United States of America

## Abstract

We discuss methods for fast spatiotemporal smoothing of calcium signals in dendritic trees, given single-trial, spatially localized imaging data obtained via multi-photon microscopy. By analyzing the dynamics of calcium binding to probe molecules and the effects of the imaging procedure, we show that calcium concentration can be estimated up to an affine transformation, i.e., an additive and multiplicative constant. To obtain a full spatiotemporal estimate, we model calcium dynamics within the cell using a functional approach. The evolution of calcium concentration is represented through a smaller set of hidden variables that incorporate fast transients due to backpropagating action potentials (bAPs), or other forms of stimulation. Because of the resulting state space structure, inference can be done in linear time using forward-backward maximum-a-posteriori methods. Non-negativity constraints on the calcium concentration can also be incorporated using a log-barrier method that does not affect the computational scaling. Moreover, by exploiting the neuronal tree structure we show that the cost of the algorithm is also linear in the size of the dendritic tree, making the approach applicable to arbitrarily large trees. We apply this algorithm to data obtained from hippocampal CA1 pyramidal cells with experimentally evoked bAPs, some of which were paired with excitatory postsynaptic potentials (EPSPs). The algorithm recovers the timing of the bAPs and provides an estimate of the induced calcium transient throughout the tree. The proposed methods could be used to further understand the interplay between bAPs and EPSPs in synaptic strength modification. More generally, this approach allows us to infer the concentration on intracellular calcium across the dendritic tree from noisy observations at a discrete set of points in space.

## Author Summary

Spatiotemporal dendritic imaging data, through fluorescent calcium indicators, opens an exciting window on computations performed by single neurons at a subcellular level. However, the analysis and interpretation of such data is challenging. The measurements are noisy, intermittent in space and/or time, and depend critically on the choice of the fluorescent indicator. Consequently, analysis is typically limited to a specific branch of the dendritic tree, neglects spatiotemporal correlations between neighboring compartments, and requires averaging over multiple trials. Here we derive a model for the spatiotemporal concentration of calcium bound probe molecules. Using state-space and optimization tools we derive a fast algorithm for estimating the most likely concentration based on the given measurements obtained from a single trial, and argue that it can provide an estimate of the fast transients of the underlying calcium concentration. In particular, our algorithm estimates the timing and amplitude of calcium transients due to backpropagating action potentials. It provides a flexible approach to inferring the structure of dendritic dynamics that are important in neural computation, but are inaccessible to direct measurement with current experimental techniques.

## Introduction

The problem of understanding the mechanisms that govern the change in strength of a synapse remains a key problem in cellular neuroscience. Fluorescence microscopy provides a way to examine aspects of the structure and specifically the function of living cells that are inaccessible to direct electrical recording. The experimenter performs optical recordings after delivering fluorescent probe molecules that translate a biological or biochemical signal into an optical output (for reviews see [1], [2]). For instance, calcium indicators are such fluorescent probes that, upon binding calcium ions, change the amount of emitted light, which can be measured with a photo detector.

The development of fast scanning multi-photon microscopy techniques has revealed that intracellular calcium concentrations play an important role in the interplay between backpropagating action potentials (bAPs) and excitatory post-synaptic potentials (EPSPs) that mediate synaptic changes through spike-timing dependent plasticity (STDP). However, the available experimental techniques still lead to noisy and spatiotemporally-subsampled observations of the true underlying calcium signals. Therefore we must use statistical methods to infer the details of the calcium transients from observed data. However, optimal spatiotemporal smoothing of the calcium profile on a dendritic tree given localized noisy measurements remains a difficult computational problem due to the high dimensionality (in terms of number of compartments) and complex structure of dendritic trees.

In this paper we present a general methodology for fast spatio-temporal smoothing of calcium signals on dendritic trees, based on single-trial experiments. We take a functional approach according to which the evolution of calcium concentration on the whole tree is determined from a smaller set of hidden variables. These govern the temporal dynamics of the calcium bound probe molecules, at small but overlapping regions of the tree, and incorporate possible concentration “bumps” due to bAPs, EPSPs or external stimulation. These bumps in the hidden states are in general rapidly increasing and slowly decreasing, and model the corresponding spatially localized bumps in probe molecule concentration due to rapid calcium transients. The calcium measurements are then expressed as linear noisy measurements of the hidden variables. Our problem then reduces to the maximum a-posteriori space-time estimation of these hidden states. Using a standard state-space approach we formulate our problem as one of optimization that can be efficiently solved if the state-transition and measurement-noise distributions are log-concave in the hidden states. In this case the problem can be solved with a cost that scales linearly with 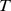 (where 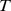 is the number of timesteps over which we would like to infer the underlying signal) using standard convex optimization techniques. These convex optimization approaches usually scale cubically with the number of hidden states 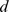, and are thus inapplicable to arbitrarily large trees. However, we exploit the localized structure that underlies our approach to show that the complexity of the present estimation algorithm also scales linearly with the size of the tree, leading to a total overall complexity that scales linearly both with 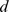 and 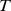.

Although related, the problem that we deal with in this paper is different from the one of extracting spikes from mesoscopic fluorescence recordings. In the latter, the data consists of images taken at a low rate from a population of neurons, and the goal is to extract a set of spike times. Several methods have been developed for this problem, such as template matching [3], supervised learning [4], particle filtering [5] and fast nonnegative deconvolution [6]. Our model is a spatiotemporal generalization of the one presented in [6], with several important differences. Our data consists of fast localized measurements at certain locations of the dendritic tree. Instead of extracting spike times, our goal is to smooth the data and estimate the full spatiotemporal profile of the calcium concentration over the entire dendritic tree, including locations at which no measurements were made. Moreover, the spikes in our case correspond to fast calcium transients due to bAPs, and apart from their timing, the estimation of their amplitude is also of great importance.

We will consider data obtained by measurements of probe fluorescence obtained with fast random-access multi-photon (RAMP) microscopy [7] from a discrete set of recording sites across a single cell. The light intensity emitted at each site is related to the concentration of calcium-bound probe molecules, and hence the underlying calcium concentration. It is important to note that we can only estimate the signal up to an affine transformation of the spatio-temporal calcium concentration. As we discuss further below, estimation of the true calcium concentration is possible, but would require further signal processing that is affected by the various noise sources. Thus we estimate the calcium concentration up to an affine transformation, which still retains all the qualitative characteristics of the spatiotemporal calcium transients. We demonstrate our algorithm with examples on artificial and real data, and discuss some different possible choices of the prior on the hidden states. This prior can be chosen to reflect our a priori knowledge about the location, amplitude, and smoothness of the calcium bumps.

## Methods

Measurements of the light intensity emitted by calcium sensitive dyes at discrete points in space and time can be used to infer the evolving concentration of calcium across the dendritic tree. Here we provide a description of an experimental situation suited for this approach, and show how it leads to a statistical model that can be used to estimate the calcium signal.

### Outline of experiment

**Figure 1:**
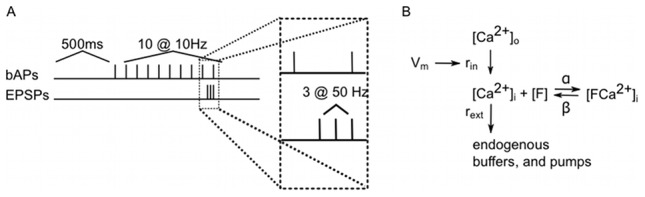
Experimental protocol and simplified reaction scheme. A: Experimental protocol. In the first protocol 10 spikes were initiated at the soma 500 ms after the start of the recording with a frequency of 10 Hz. In the second protocol, the last action potential was preceded by a series of 3 EPSPs evoked at the dendrites, starting 50 ms before the last spike at a frequency of 50 Hz. B: The relationship between membrane potential and fluorescence. Changes in membrane potential affect the rate of 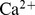 influx into the cell via voltage gated calcium channels. Intracellularly, 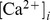 is sequestered by endogenous 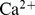 buffers, as well as by the optical probe, 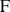. A change in the relative concentration of 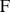 and 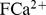 results in a measurable change in fluorescence.

Although the statistical methods we present are general, we demonstrate them in the context of a particular experiment. It has been assumed that action potentials that backpropagate from the soma into the dendritic tree play an important role in learning [8]. We examined how bAPs interact with synaptic input in pyramidal cells from the CA1 region of the rat hippocampus *in vitro*, in acutely dissected hippocampal brain slices. A schematic of the stimulation protocol is shown in Fig. 1a: 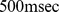 after the start of the recording ten action potentials (APs) were initiated at the soma of the pyramidal cell at 10 Hz. In some experiments, one of the 10 evoked APs is paired with a series of 3 synaptic EPSPs at 50 Hz. These EPSPs were evoked starting at 50 ms before the selected action potential by a bipolar extracellular stimulating electrode placed at 20–50 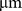 from the dendrite of the recorded cell, approximately 250–300 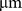 from the cell body layer.

Each evoked AP backpropagated into the dendritic tree. These bAPs resulted in a transient increase in intracellular calcium concentration, which has been previously shown to result from increased conductance through voltage-dependent calcium channels [9], [7]. The goal of the experiment was to study the effects of the pairing of a bAP and EPSPs on the spatiotemporal structure of the calcium concentration across the dendritic tree. Each recording lasted for approximately 6 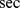.

Recordings were performed using 3D RAMP microscopy, which uses acousto-optic deflectors (AODs) for high-speed recording within a volume of live brain tissue [7]. Recording sites were chosen by the user, and their location was fixed during a recording session. A recording cycle took only 20 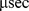 which allowed a functional scan consisting of 30 arbitrarily positioned recording sites to be made at a 1.6 kHz sampling rate.

### Construction of the statistical model and the inference algorithm

#### Calcium-bound probe molecules and the emitted light signal

The concentration of calcium-bound probe molecules can be used as a proxy for the concentration of calcium in the system. A simplified reaction scheme describing calcium binding is shown in Fig. 1b. As probe molecules bind to calcium quickly, we assume that during fast transients, the change in concentration of fluorophore bound to calcium 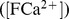 is proportional to the calcium concentration ([ 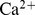 ]) times the concentration of free fluorophore at this location. This relationship holds as long as probe molecules are not saturated with calcium. Our optical probe, Oregon Green BAPTA-1, has a high affinity to calcium, due to its fast binding rate, 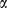, and slow unbinding rate, 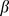, leading to decay rates on the order of 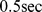. As a result, the fluorescence response from a fast voltage transient, like that of a bAP or an EPSP, rises effectively instantaneously, and decays slowly [10], [11]. Thus the fluorescent signal provides a temporally filtered representation of the underlying intracellular calcium concentration. This complicates the interpretation of the recordings. Our goal will be to reconstruct the spatial and temporal structure of bound probe molecule dynamics. We therefore first describe how the emitted light intensity is related to the calcium concentration at a location within the cell.

The light intensity emitted at location 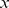 at time 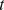, 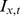, is directly related to the concentrations of bound probe molecules by
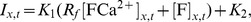
where 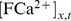 and 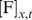 are the concentration of *bound* and *free* probe molecules respectively, at spatial location 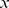 and time 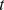. The scalar 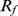 represents the relative fluorescence of the bound fluorophore to free fluorophore (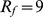 for the dye used here [12]). The constants 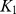 and 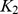 represent the light intensity of free probe molecules and autofluorescence, respectively.

Assuming that probe molecules are conserved locally, i.e., 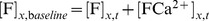, we obtain
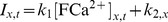
(1)where 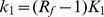 and 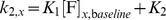. Therefore fluorescence, 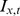, is affinely related to the concentration of calcium-bound probe molecules when these are not saturated. To simplify our analysis we assume that Eq. (1) is deterministic and that all the noise in our model comes from the recording procedure, which will be described below. By defining the baseline fluorescence at location 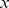 as 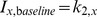, we have that 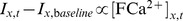.

#### The signal of interest

The fast calcium signals in the tree, in our case, are due to a bAP that results in a transient increase in the concentration of bound probe molecules. High affinity calcium dyes, such as the Oregon Green BAPTA-1 (OGB-1) used here, bind calcium with a very fast onset and also have a much slower dissociation rate. Neglecting spatial correlations and diffusion terms, we can model the bound probe molecule dynamics as
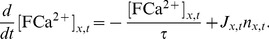
(2)Here 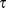 is the time constant that determines the rate at which probe fluorescence molecules unbind from calcium. The term 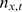 represents the times at which the sequence of bAPs reaches the location 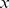 and can in this case be written as the sum 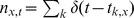 of Dirac delta functions, where 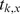 represents the time at which the 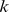-th bAP reaches location 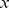. We will generalize this assumption below. The 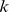-th bAP at location 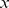 results in an instantaneous jump of size 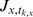 in bound probe molecule concentration, followed by an exponential decay to a baseline concentration at rate 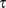. For a high affinity dye a fast calcium influx leads to a 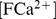 transient with rapid onset. Moreover, from the reaction scheme of Fig. 1b the amplitude of this transient depends also on the baseline concentration 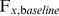 of the optical probe at each location 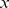.

This baseline probe molecule concentration typically varies across the cell and is difficult to control *a priori*. For instance, probe molecule concentrations are typically higher in the apical dendrite and near the soma, resulting in the higher luminosity of these regions. This variation may be due to nonuniform concentration of basal calcium across the cell. Also contributing may be limitations of the experimental setup, such as non-uniform laser illumination across the microscope's field of view or non-uniform volume of fluorophore excitation, due to thin processes that extend beyond the focal volume. Moreover, recording sites are sometimes placed slightly off a dendrite. To account for such variability in baseline fluorescence, we assume that such non-uniformities are primarily due to the experimental limitations which affect the number of probe molecules being excited. As a result, and based on the dynamics of calcium binding, we expect that during fast transients 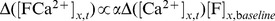, where 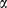 is the binding rate of our optical probe. Implicit in this relation is the assumption that the concentration of probe molecules is away from saturation, and that the fast calcium transient is mainly due to the bAP. Therefore, Eq. (2) can be rewritten as
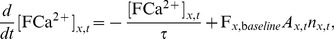
(3)where 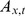 represents (up to a multiplicative constant) the amplitude of the calcium transient at location 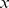 and time 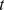.

Based on the above discussion, we make an important conclusion: The *relative* change in bound probe molecules is reflective of the transients in calcium concentration. Hence, by Eq. (1), we need to estimate *relative* changes in light intensity at a given location, as a proxy for transients in calcium concentration. Dividing Eq. (3) by 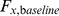 we get
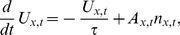
(4)where 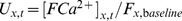. As discussed above 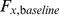 cannot be easily estimated. However, from our measurements we have access to 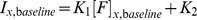. To be able to compare the calcium concentration at different locations of the tree, we need that approximately 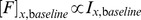 for all the locations in the tree. This assumption is expected to hold if 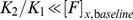 for all locations, which is expected since the autofluorescence signal is in general weak for high affinity calcium indicators. Therefore from now on we assume that the relative change in bound probe molecules 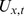 is approximately proportional to the 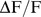-transformed emitted light intensity, with a constant of proportionality that is the same across the tree:
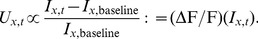
(5)Note that this resulting 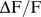 transformation is a standard heuristic used in the analysis of fluorescent recordings [9], [8].

#### The recorded signal

The signal, 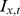, is weak, and is measured using photomultiplier tubes (PMTs), sensitive light detectors which contain several amplifying stages. Briefly, an electron entering one stage will produce a random number of outgoing electrons. If 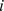 is the number of incoming electrons at a stage, the number of outgoing electrons, 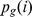, is random. The mean of 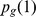 is the gain of the stage. For instance, 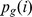 could be a sum of 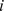 numbers drawn independently from a Poisson distribution with mean 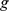 [13]. The output of an eight stage PMT is then an eight-fold composition of 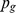 with itself. The noise in the output of the PMT can therefore be highly non-Gaussian, with a distribution that is determined by the strength of the signal 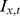. In particular, at lower light levels 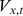 approximately follows an exponential distribution (data not shown). At higher light levels, the distribution is approximately Gaussian [14], [13]. The photomultiplier statistics can be fit with a gamma distribution with activity dependent parameters:
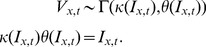
(6)When we fit our data to this model we obtained 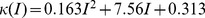 (data not shown). Note however that the signal of interest is not 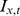 itself but its relative change (see Eq. (5)). If 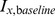 is known, then the scaled random variable 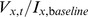 is distributed according to 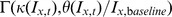. In that case the variance of the relative change in light intensity remains approximately constant with small changes in 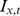 relative to 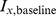. The skewness and kurtosis of the distribution are given by 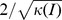 and 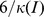 respectively. As 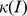 becomes large, they become very small and the resulting distribution is approximately Gaussian [15]. This happens at locations with moderate or high baseline activity. As a result we first assume that the signal is sufficiently strong and the noise is Gaussian, so that 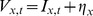 for a Gaussian random variable 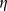. We relax this assumption subsequently. However, we always assume that the expected value of the measured voltage is proportional to the true light intensity 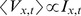.

#### A general statistical model for the spatiotemporal calcium signal

Using Eq. (4) for the dynamics of relative bound probe molecules in the presence of incoming calcium spikes, we have that
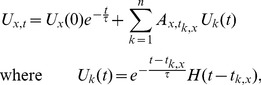
(7)where 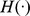 denotes the Heaviside function and 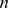 denotes the number of spikes. If the time at which a bAP is initiated is known, then the only parameters that need to be estimated are the amplitudes 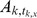. We can assume that a bAP propagates through the tree instantaneously, so that 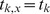 for all locations. Under this assumption amplitudes can be easily estimated using maximum likelihood techniques, with the added constraints that the inferred amplitudes are nonnegative. We will fully describe these techniques in a more general setup below.

More generally, the timing of the APs may not be known or not the same for all the locations in the tree and must be inferred from the bound probe molecule trace. This is a challenging problem in itself [6]. Moreover, the above approach neglects spatial correlations caused from the backpropapation and possible calcium diffusion mechanisms. As a result, the calcium profile at each location is treated independently and cannot be used to make predictions for neighboring, possibly non-imaged locations. Instead of analyzing the recorded signals from each location separately we would like to take advantage of the fact that the recording from one location may provide information about the signal at neighboring locations. We next describe a statistical approach that allows us to perform spatial and temporal smoothing concurrently.

To address the issue of spatial correlations, we model the relative concentration of bound probe molecules at location 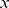 of the dendritic tree at time 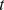 as a linear combination of spatial functions whose weights are allowed to change with time,
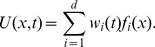
(8)The spatial functions 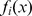 are bump functions which are smooth, nonnegative and local (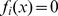 for 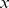 further than some distance from the “center point” 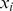 of the 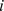-th bump). These functions serve to spatially smooth the inferred signal, since 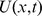 cannot vary more quickly in space than 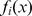; by changing the number of bumps 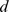 and their smoothness we can modify the overall spatial smoothness of 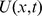. There are a number of possible interpolation approaches, and we chose to use B-splines [16]. Other choices include gaussian bumps or diffusion kernels [17] that preserve continuous differentiability at the tree branch points. However, the exact choice of basis functions did not considerably affect our results, as long as these functions are smooth and are placed approximately uniformly along the tree.

As the spline basis functions are typically defined on a line, here we use a suitable generalization to trees. Splines are defined on subdomains of the tree defined by the cell shape. This tree therefore needs to be partitioned. We chose to define the partition using the topological distance from the soma, i.e., the distance from the soma along the tree. Thus all points that lie within a given distance from the center of a spline function belong to the same subdomain. Picking this distance is important because a very large/small value can lead to significant under/over-fitting of the data. In the Results section where we apply our algorithm to experimental data, we propose a data-driven method for determining this parameter.

We discretize the cell into 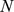 compartments so that the 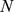-dimensional state vector of normalized bound probe molecule concentration at each location on the cell can be written in matrix-vector form as
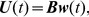
(9)where 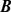 is an 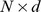 matrix whose 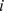-th column is a vector obtained from the spatial discretization of 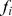, and 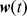 is the column vector of the activation variables at time 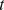, and has length 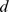.

We assume the same temporal dynamics as in Eq. (2), but now for the weight variables 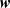, which are assumed to evolve according to
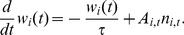
(10)Because of linearity, 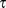 has the same interpretation as the time constant that determines the rate at which probe fluorescence molecules unbind from calcium, and 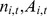 have similar interpretations as before. After discretizing time, and introducing 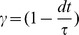, Eq. (10) can be approximated by
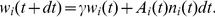
(11)In this discretized representation 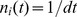 when 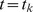, and 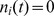 otherwise. In the general case, where the timing of the spikes is unknown, we replace the product of 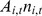 with a nonnegative random variable 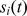.
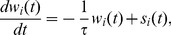
(12)This model is a spatiotemporal generalization of that discussed in [6].

There are two essential differences between the model described here by Eqs. (8)–(12) and the motivating model described in Eq. (7): estimation of spike times, and incorporation of spatial correlations. In the present model the input, 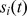, is *not* assumed to be a sum of delta functions with unknown weights occurring at known times. Moreover, as we will see below, the inference will be done at once for the whole tree and *not* individually and independently for each location. Thus, this statistical approach is more general since the algorithm is capable of finding spike times, and more biologically accurate, since it allows for spatial correlations for nearby compartments.

We use a Bayesian approach, and start by specifying a prior distribution on 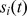, and hence construct a prior distribution on the hidden weights 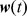 (and consequently on 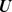
(t)). For example, if we know that calcium influx is small at certain times 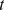 or at certain locations 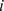, then we can choose the prior variance and mean of the corresponding 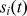 to be small. Local correlations in 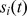 may also be incorporated to increase the smoothness of the state vector 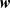. The prior on 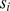 can also be interpreted as an autoregressive prior on 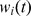 which serves to temporally smooth the inferred signal, on a scale set by the time constant 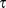. This time constant 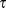 could also depend on the spatial variable 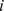 (modeling different 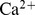 buffer constants at different locations in the tree) without significantly changing the presentation below.

Now taking into account the dynamics of probe molecules (see Eqs. (1),(4) and (5)), the emitted light intensity can be written as
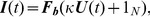
(13)where 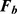 is a diagonal matrix where the non-zero entries denote the probe molecule baseline concentration, 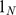 is a column vector of ones with length 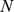 and 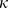 is a scalar constant, related to the proportionality constant in Eq. (5), and is assumed to be constant throughout the tree. Note that our signal of interest 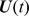 and the emitted light intensity 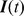 are related through an affine transformation.

We have access to 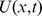 through spatially localized noisy measurements. In each measurement, we image a single compartment of the tree and at each time 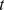, 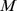 such measurements are performed. Let 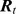 be a 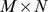 matrix where each row has one entry equal to one, corresponding to the compartment being imaged, and the rest equal to zero. Assuming additive noise, the measurements can be expressed as
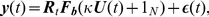
(14)The noise 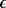 is assumed to be spatiotemporally uncorrelated, although this could be generalized to the case that the covariance matrix of 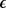 has a local structure. Note that 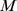, the number of measurements per time step, can easily change with time. The matrix 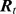 can represent more general linear measurements. Initially we assume noise is additive for notational simplicity. We show below that more general forms of noise can be modeled within this framework.

After discretizing time, Eqs. (8) and Eq. (14), correspond to the state-space model
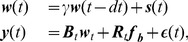
(15)where 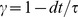 and we abbreviated 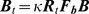 (with dimensions 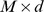) and 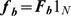. This completes the specification of the model.

We emphasize again that Eq. (14) provides a biophysically inspired statistical model. As earlier, we aim to approximate a signal proportional to the bound calcium concentration across the dendritic tree. Note that certain parameters in the model such as the time constant 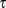 do have direct physical interpretations

#### Computing and optimizing the log-posterior

We would like to compute the maximum a-posteriori (MAP) estimate of the relative spatiotemporal concentration of calcium-bound probe molecules 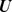, given the sequence of observations 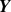. Here 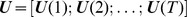 and 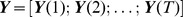 denote the vectors with the underlying calcium concentrations and measured light intensity at all times, and have lengths 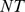, 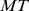 respectively. From Eq. (9) it follows that
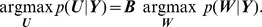
Thus we will focus on finding the MAP estimate of the activation profile 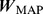, where 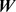 is a vector of length 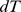 defined similarly to 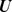 and 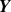. Using Bayes rule and the state-space nature of the model we can compute the log-posterior directly [18], [19]:
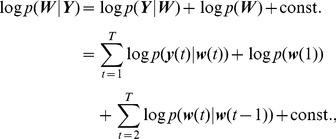
(16)where the constant does not depend on the 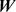 and thus can be ignored. The log-posterior 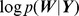, can be maximized efficiently if this function is concave in 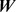. Since 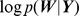 is a sum of terms of the form 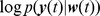 and 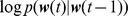, it is sufficient that these observation and transition densities be log-concave, i.e., that 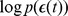 and 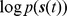 be concave in 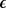 and 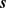 respectively. This means that other log-concave noise distributions can be used in our framework, not just additive Gaussian noise.

Additionally, since the calcium concentration is nonnegative, we have to ensure that all the activation variables, as well as the bumps 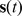 are nonnegative. These additional constraints can be expressed 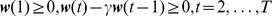, where the inequalities are in vector form, i.e., they have to hold for all the entries. Therefore our optimization problem can be written as
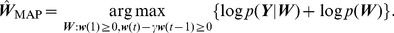
(17)


In the Supporting Information we describe the technical details of a fast method for the solution of Eq. (17). Briefly, we construct a log-barrier method for the incorporation of the non-negativity constraints [20]. In general, barrier methods are iterative procedures that converge within only a few iterations, but at cost per iteration 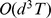 in time and 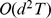 memory requirement. The so called “big O” notation for the time cost 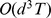 is used to denote that every iteration requires a number of operations that scales linearly with the length of the experiment 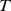 and cubically with the size of the imaged tree 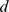. We see that for large trees this cost can be prohibitive. However, by exploiting the tree-structure and the spatially localized nature of the measurements, we show that the cost per iteration can be dropped to 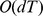 in terms of both time and space, making our method applicable to arbitrary large dendritic trees. Details can be found in the Supporting Information.

### A particular example

We discuss a particular example of choices for the prior and noise likelihoods that are realistic and also ensure log-concavity. As a first example, we consider the case where the 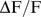-normalized measurements 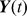 follow a Gaussian distribution with variance 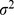. The simulated calcium bumps have deterministic fixed amplitude 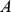. We have
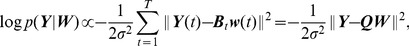
(18)with 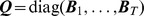, the measurement matrix at all times. The imaging locations at each time can change.

The prior on 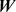 depends on the temporal statistics of calcium activation and its amplitude at different locations of the dendritic tree. For simplicity, the hidden state bumps are assumed to be independent in time and space. Note that this assumption of independence applies to the hidden states. The inferred concentrations of bound calcium are obtained from the hidden states by interpolation using splines. As a result, the concentrations at neighboring compartments on the tree will be significantly correlated. The extent of these correlations is determined by the structure of the splines used in the interpolation. Nevertheless, the assumption of independence is a simplification, since a bAP is expected to increase the intracellular calcium concentration throughout the tree at approximately the same time. However, this assumption allows for a fast implementation of our algorithm, and in practice does not affect its performance.

A reasonable intuitive choice for the bump prior would be a marked point process [21], i.e., a sequence of pairs of spike times and amplitudes. However, since the latter is not in general log-concave, we can approximate it (as in [6] for the case of a Poisson process) with an exponential distribution with a desirable rate. The exponential distribution corresponds to a sparse, nonnegative prior, that also retains the desired property of log-concavity. The prior 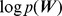 has the initial value 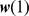 chosen to be an exponential with parameter 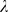. For the state transitions, we let the differences 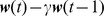 follow an exponential distribution with mean 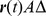, where 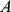 is the amplitude of the bump (assumed constant here), 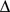 is the time discretization step 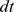 and 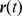 is a vector that depends on the possibly time-varying, rate. Therefore,
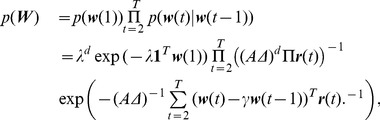
(19)where 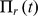 denotes the product of all the entries of the vector 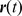, and 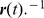, denotes the vector obtained by inverting each entry of 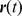. Taking the logarithm and removing the additive terms that do not depend on 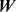 we get,
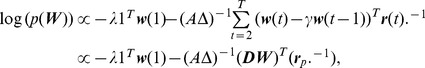
(20)with 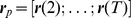, and 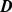 is an appropriate matrix such that 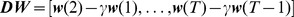.

Putting everything together we seek the MAP estimate
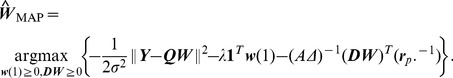
(21)We can find this estimate efficiently using the method described in the Supporting Information. Note that the total number of parameters to be estimated is 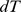, where 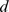 is the number of hidden weights 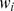, and 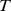 is the number of timesteps.

## Results

We demonstrate our approach using data generated by numerically simulating calcium transients on a dendritic tree, and then continue with the analysis of experimental data.

### Application to simulated data on a real dendritic tree

We apply the developed methodology on a reconstructed dendritic tree from a rat CA1 pyramidal cell. The tree was discretized in 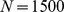 compartments and 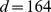 hidden variables and spline functions were used to fit its dynamics. We simulated a series of three bAPs that propagate instantaneously and unselectively throughout the tree with amplitude that decreases exponentially with distance from soma. The prior on the amplitude of the hidden states due to the bAP was estimated from the transient pattern induced from the bAP, with methods that are explained below. We assumed that the calcium-bound probe molecule concentration relaxes with a single time constant that is estimated from the data and that the noise likelihood is known. For the noise likelihood we used a gamma distribution with parameters that depended on the true underlying value according to the statistics of the PMT used in the experiment (see Eq. (6)). At every time step 200 randomly chosen points are measured.

**Figure 2:**
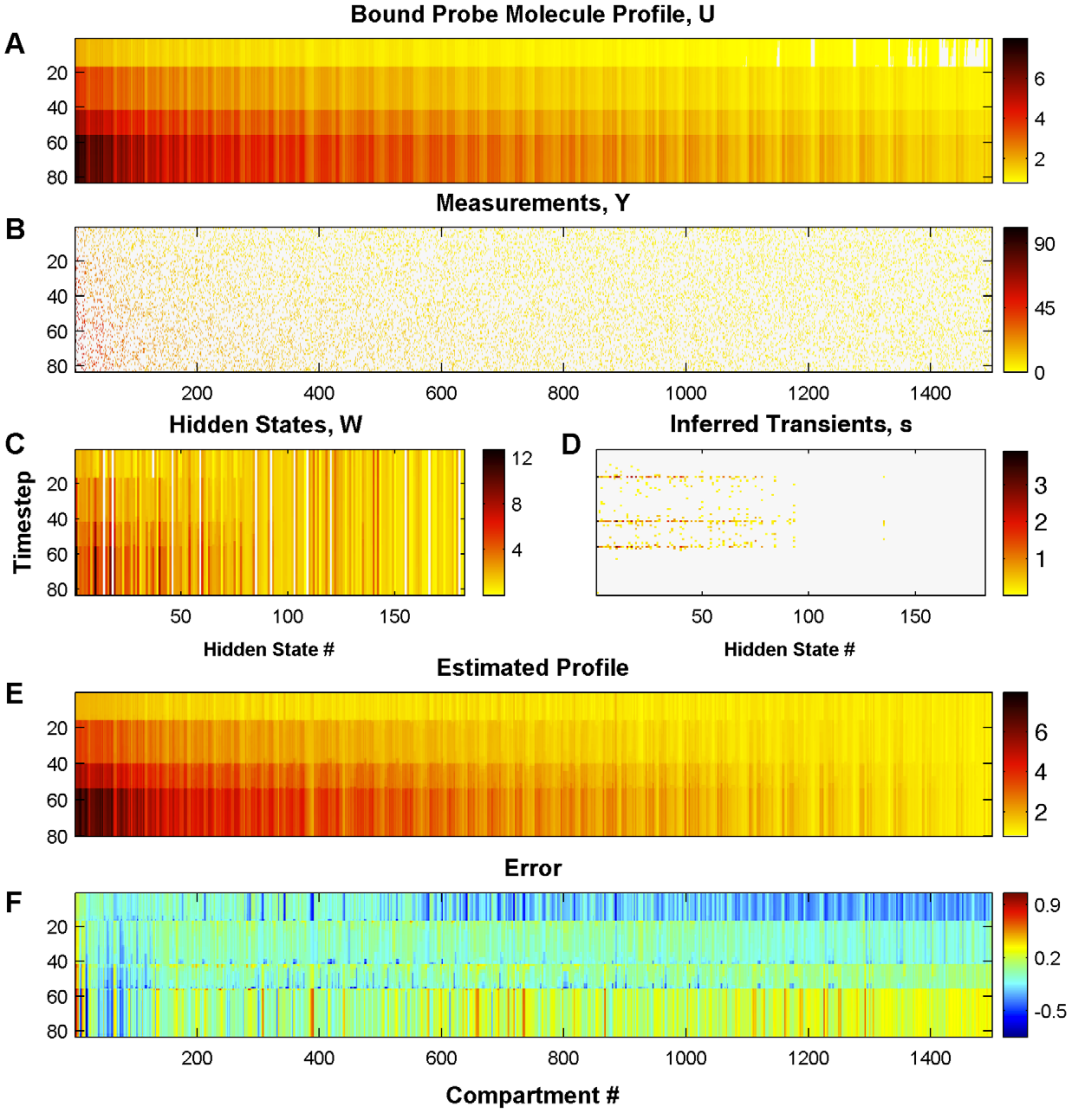
True and estimated probe molecule concentration and inferred transients. A: True calcium traces of a simulated experiment. Three bAPs were simulated at timesteps 20, 50 and 66. B: Measurement locations and values at each timestep. The dark pixels correspond to locations that were not measured at the specific timestep. C: Inferred weights of the hidden states 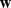 (top) D: Inferred transients of the hidden states 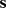 (bottom). E: Estimated calcium traces for all compartments. F: Error between estimated and true profile 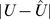. The algorithm correctly infers the timing of the bAPs and estimates their amplitude (see also Fig. 3). The colorbars in the panels A and E are the same.

**Figure 3:**
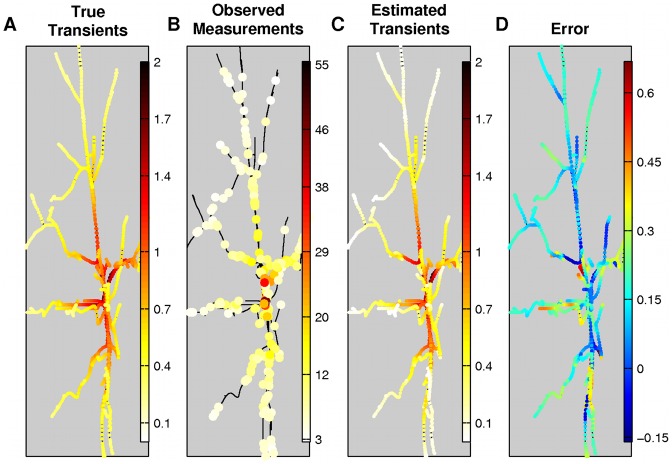
True and estimated amplitude of the second transient along the tree. Estimated transient due to the second bAP in the simulated experiment. A: True bound probe molecule transients. The transient occurs in the whole tree with an amplitude that decreases with the distance from the soma. B: Noisy measurements and their locations at the time of the bAP. C: Estimated transients. D: Ampltiude error (true - estimated). The amplitude is mostly underestimated due to the bias-variance trade-off. The colorbars for the first and third columns are the same.

Fig. 2 shows the true bound probe-molecules profile, the observed measurements, the inferred weights and transients, and the resulting estimated profile and error 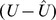. The algorithm correctly predicts the timing of the bAPs and estimates the true bound probe molecules concentration. Fig. 3 shows the amplitude of the second transient, the measurements right after the spike occurrence, the estimated transients and error. As discussed earlier, these transients estimate up to an affine transformation the underlying calcium transients. A close inspection reveals that in certain cases the algorithm underestimates the transient amplitude. The reason for this is that our algorithm acts as a shrinkage estimator due to the exponential prior [22]. The prior in our analysis is included to provide more robust estimates in the presence of high noise. Because of the bias-variance tradeoff [23] a robust estimator (i.e., with low variance) can be biased in the presence of high noise, as is in our case. If no prior is incorporated, then our estimate can in principle be unbiased (e.g., the least squares estimator in the presence of Gaussian noise), but it would also have a large variance (data not shown). This leads to over-fitting and is undesirable since the same underlying calcium concentration can lead to very different estimates, due to different noise realizations.

Sample code to generate Figs. 2 and 3 as well as a movie that shows the spatiotemporal profile of the calcium in the tree can be found at http://www.stat.columbia.edu/~eftychios/Home/Calcium_Smoothing.html

We also tested the effect of the minimum topological distance between imaged positions. We used the same setup as above, but imaged the signal at 100 locations at every time step. Additionally, at each repetition we imposed a minimal topological distance between the imaged locations. Since the units are arbitrary here the exact results are not reported. However, for small values of the minimal distance the error grows slowly with the minimal separation between the imaged locations. Large errors are observed when the minimal distance between imaged locations becomes comparable or larger than the distance between centers of the interpolating spline functions. In the next section we present an adaptive method of determining the smoothing spline basis based on the locations of the measured locations.

### Application to real data

**Figure 4:**
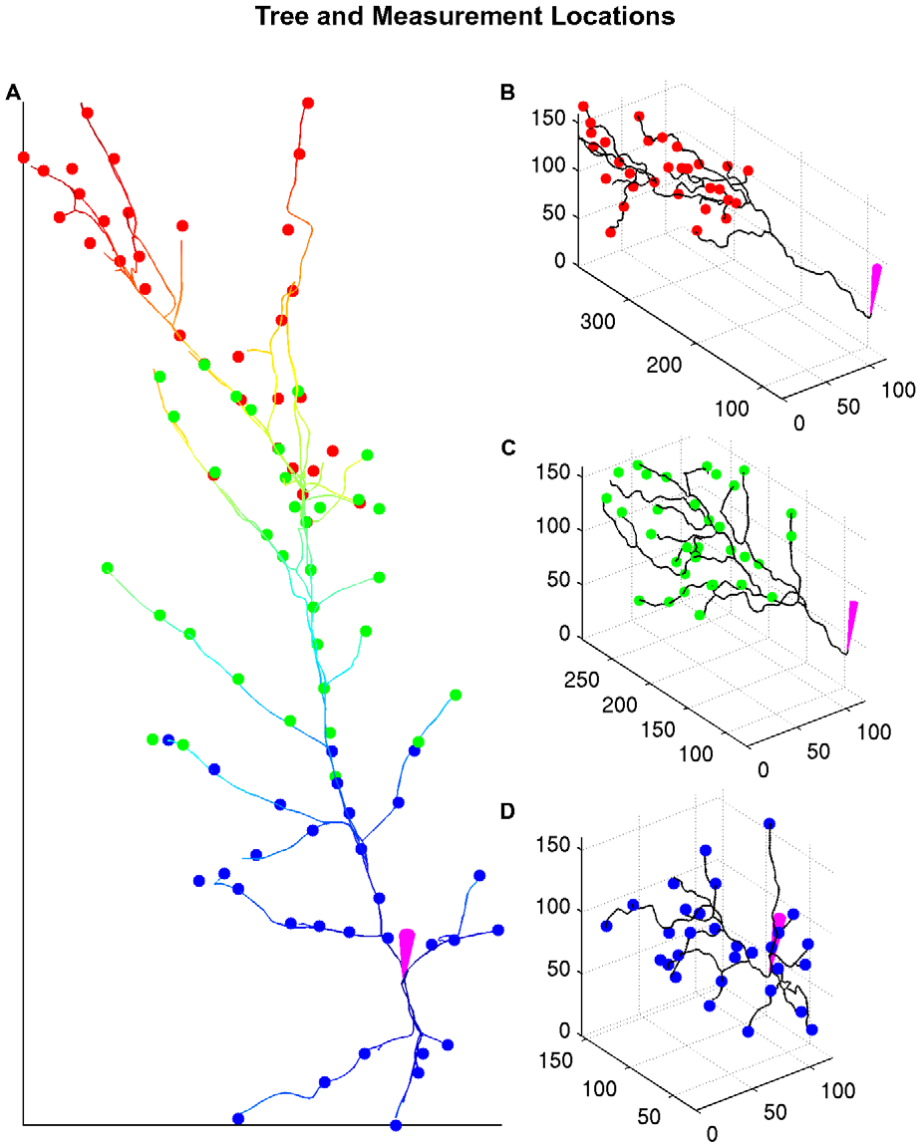
Tree and measurement locations without the non-imaged dendrites. A: Tree projected in the x-y plane and measurement locations. Each compartment is also color-coded according to its distance from the soma. The different colors in the measurement locations correspond to the different imaging sessions. Right: Detailed subtrees in three dimensions for the three different imaging sessions. B: Subtree A, recording locations in the apical tuft. C: Subtree B, recording locations around the apical dendrites. D: Subtree C, recording locations around the soma and basal dendrites. All the units are in 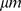. In all panels the purple electrodes point at the soma.

The algorithm was tested on calcium imaging data from a CA1 pyramidal neuron obtained with a fast three dimensional optical scanner. The details of the scanner are given in [7]. The reconstructed neuron consisted of 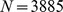 compartments. Recordings were made at 97 different locations in 3 different sessions. Every location was imaged at approximately every 0.6 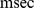. For simplicity, the dendrites that did not contain any recording locations were removed from the tree graph (using the TREES toolbox [24]) when analyzing the data. The resulting truncated tree and the measurement locations are shown in Fig. 4. The three subtrees A,B,C are shown in panels B,C,D of Fig. 4 respectively. Subtree A corresponds to the imaging session on the dendritic tuft of the neuron. Subtree B corresponds to the recording sites in the apical dendrites of the neurons and finally subtree C corresponds to the recording sites in the basal dendrites and around the soma.

For each pair of imaging data we estimated one set of model parameters, so that the results of the imaging algorithm on the two different protocols could be compared. The measured data was normalized using the 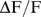 transformation (see Eq. (5)) before being analyzed. The data was analyzed with the same set of model parameters for the two different experimental protocols and separately on the three different subtrees obtained from the three different imaging sessions.

#### Estimation of the model parameters

Our model has several parameters that have to be estimated before we apply our smoothing algorithm. These are the baseline probe molecules concentration, the time constant of calcium unbinding and the noise statistics. Below we describe simple heuristic methods to estimate the model parameters. In practice more sophisticated and powerful methods can be developed such as Monte Carlo estimation methods (e.g. via the Expectation Maximization algorithm) [25]. However, we saw that the results of our algorithm are qualitatively robust with respect to the various parameter values. We also discuss how we chose the appropriate spline matrix for each subtree and the prior on the transient amplitudes.

#### Baseline probe molecule concentration

The baseline fluorophore concentration 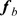 was estimated by taking the time average of the measurements over the first 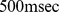 on all the imaged locations. This corresponds to the maximum likelihood estimate of the baseline activity both under Gaussian and under gamma measurement noise.

#### Time constant estimation

Next, the decay constant of the recorded signal, 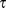, through its discretized version 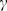, was estimated: First the measured calcium traces were passed through a low-pass, moving average filter (with 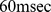 width) to eliminate the high frequency noise effects (shot noise). After that, the discretized time constant was estimated by fitting a first order autoregressive model to the filtered data which was first normalized to have zero baseline.
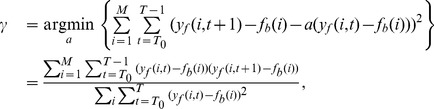
(22)where 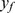 is the filtered measured calcium trace, 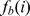 is the baseline concentration at location 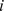, and 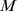 is the number of recording locations. 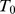 was chosen 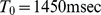, i.e., shortly after the last bAP. This estimator gave 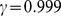, or 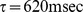. The time constant was estimated separately for each subtree and the estimates were approximately equal.

#### Spline matrix and amplitude prior

The state transition (amplitude) prior was considered to be an exponential distribution, as discussed in the Methods section. There a marked point process with rate 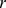, mean amplitude 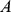, and discretized in time at resolution 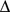 can be approximated by a log-concave exponential distribution with parameter 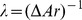. Therefore we need to determine the amplitude of the hidden states at each location and the rate which is time varying. Note that in general the mean amplitude can be time varying, but we approximate it as constant with time. The B-spline matrix 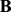 and the amplitude of the bump were estimated via an alternating procedure as follows: The basis functions were third order polynomials whose centers were placed uniformly on the tree, and the only parameter required for determining the splines was the topological distance (i.e., number of compartments), between two neighboring center points. The choice of this distance is important since a large distance can lead to a less flexible model, whereas a small one can cause over-fitting. We picked the maximum distance that allows the model to fit the estimated amplitude of the calcium bumps. Choosing a smaller distance gives more degrees of freedom to the system and can potentially lead to overfitting. Note that the prior amplitude pertains to the hidden state vectors 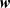 and thus cannot be readily estimated from the data. We first obtained a crude estimate of the amplitude of the normalized calcium transient as
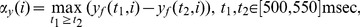
(23)i.e., the maximum difference after the first bAP, measured in the filtered traces. Then to determine the amplitude for the hidden states 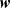 and the B-spline matrix we proceeded as follows: For any topological distance 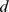 between two neighboring spline function centers, a different basis matrix 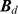 was constructed and the prior amplitude vector 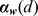 on the state variables 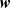 was given by the solution of the quadratic program
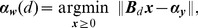
(24)This provided the non-negative amplitude parameters 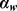 of the exponential priors that can best approximate 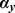 (the estimated calcium transients) for the given topological distance 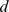. If this approximation had a relative error larger than 0.1, we decreased the topological distance 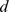 and repeated the same procedure. The actual used topological distance 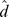, the splines matrix 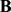, and amplitude prior parameters 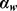 were picked as
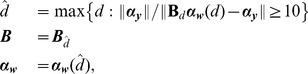
(25)i.e., we picked the maximum distance, and hence the smaller number of hidden variables, that allow for a satisfactory interpolation of the estimated calcium transients from the hidden state amplitude prior parameters. The rate 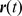 of bumps was time varying and equal for all the states, given by
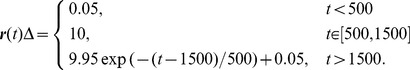
(26)The rate starts with a low value at the first 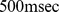 since we do not expect any spikes there. Then, it is set at the value 10 since we expect 10 bAPs during the one-second excitation window. After that, the rate decays smoothly back to the initial value. As we explain further below, this smooth decay prevents the algorithm from inferring a non-existent spike at 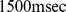 due to the temporal discontinuity of the rate. Note that with this model, the exact spike times are considered unknown and are also to be estimated by the algorithm.

#### Noise statistics

Finally, the noise likelihood at each measurement location was assumed to be independent and Gaussian with zero mean, and variance estimated directly from the measurement traces. To find the noise statistics we subtracted the filtered traces 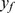 from the 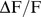-normalized data and then fitted a Gaussian distribution on the residual. Although this is a simplified approximation, in most of the cases this residual could be fit satisfactorily with a zero mean Gaussian distribution (data not shown).

### Spatiotemporal smoothing of calcium-bound probe molecules

**Figure 5:**
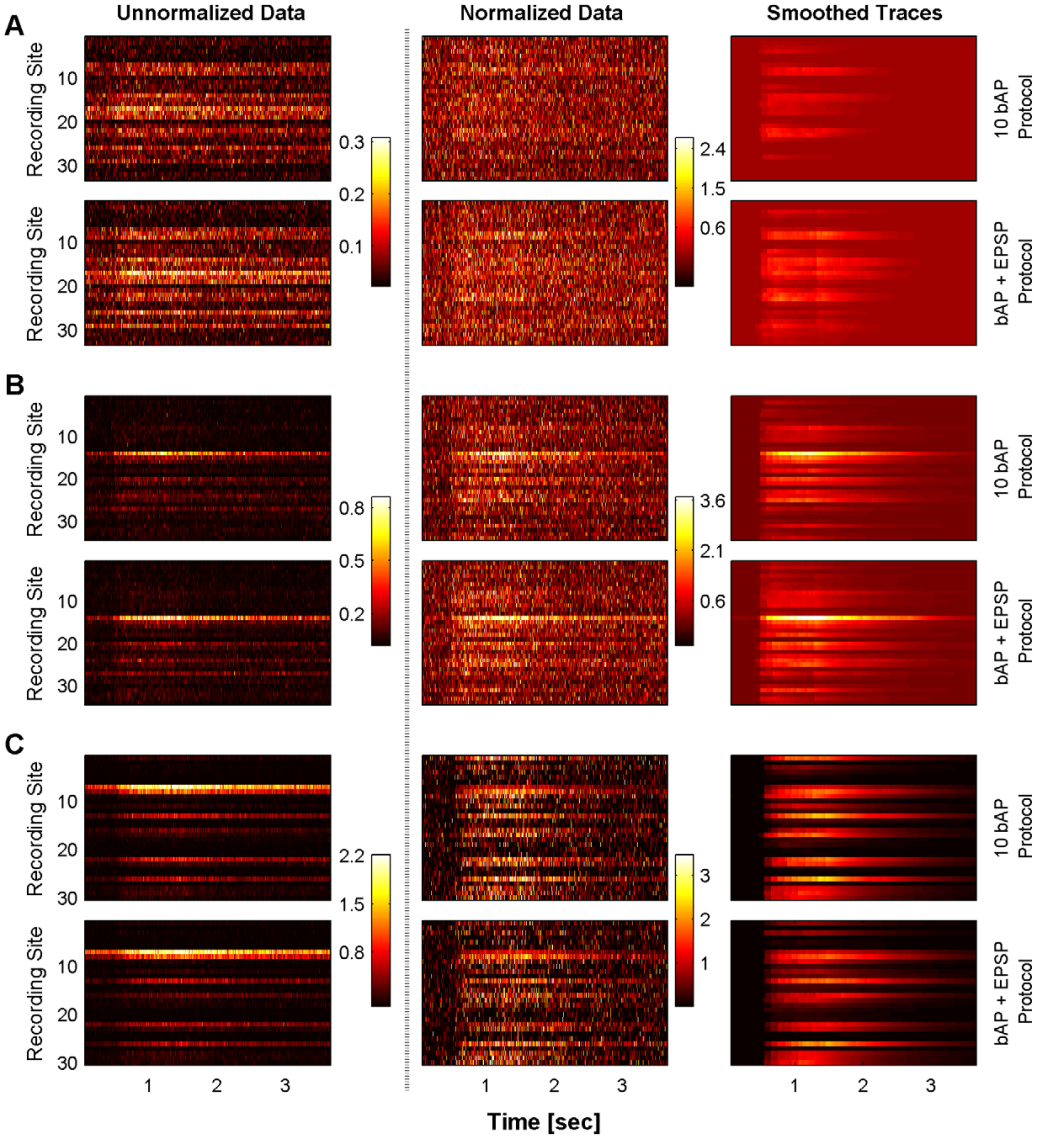
Observed and smoothed data at all the imaged locations for both experimental protocols. For each subfigure: First column: Raw data 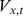 (unnormalized). Second column: Raw data 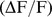 normalized 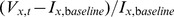. Third column: Smoothed data at measurement locations 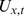, estimate of the relative concentration of calcium-bound probe molecules. Top row: 10 bAP protocol. Ten spikes were applied at the neuron soma at times 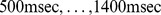. Bottom row: 10 bAP+3 EPSP protocol. 3 additional EPSPs were applied at times 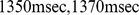 and 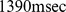. A: Subtree A (tuft), B: Subtree B (apical dendrites), C: Subtree C (soma and basal dendrites). The algorithm smooths the data and provides an estimate of the underlying calcium concentration. Only the imaged locations are shown for comparison purposes. The inferred spikes are visible from the discontinuities in the third column. Spikes are not inferred in all locations indicating the selective propagation of calcium in the tree.

Fig. 5 shows the measured data, and the inferred traces at all measurement locations, for all three subtrees and the two different experimental protocols. The first column shows the measured raw data. The last two show the data and the inferred traces in the 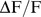 domain, i.e., the input and output of the algorithm. Based on the discussion of the Methods section these inferred traces are an estimate of the calcium bound probe molecules up to an affine transformation, and thus their transients are indicative of the calcium transients. From Fig. 5 we see that the algorithm detects the spikes in the window 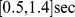 (visible especially in the last column), and that the calcium concentration in general decays with the distance from the soma (see the color bars in the right column), a fact that has been reported in many studies (see e.g. [26], [27]). In the first column of Figs. 5B and 5C we observe that the measured light intensity in some locations is significantly larger in a few points than the rest. These points lie in the dendritic trunk and also exhibit a larger baseline intensity. However, as seen from the last column, spikes are detected in most of the measurement locations, but not all of them, indicating the selective propagation of the bAPs. The results can best be viewed in video format, where the spatiotemporal probe molecule profile can be examined in the whole tree, and not just in the recording sites. Such movies can be found online at http://www.stat.columbia.edu/~eftychios/Home/Calcium_Smoothing.html.

**Figure 6:**
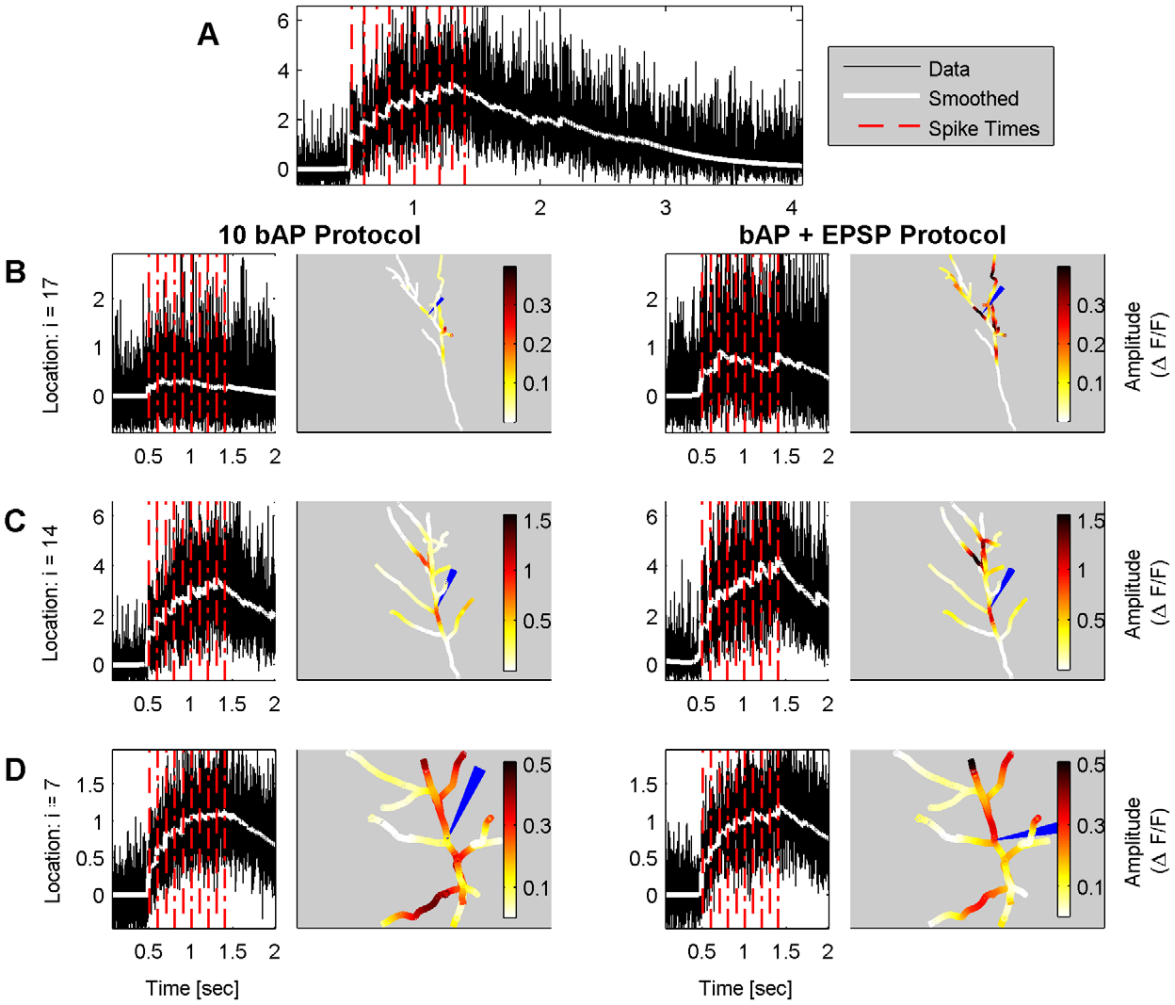
Detailed probe molecule traces and inferred transients. Detailed probe molecule traces for one location of each subtree (marked with the blue electrodes on the right columns) and profile of the amplitude of the last transient inferred in the whole subtree. A: Detailed measured and inferred trace for the whole length of the experiment at the marked location of subtree B. At each of the other rows: Detailed measured data and smoothed traces (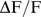 normalized) for two different locations (top and bottom row. The red dashed-lines correspond to the timing of the bAPs. The algorithm reduces noise considerably, accurately infers the timing of the bAPs and provides an estimate of their amplitude. The second and fourth columns provide an estimate of the amplitude of the last transient. Left: 10 bAP protocol. Right: 10 bAP+3 EPSP protocol. B: Subtree A (tuft), C: Subtree B (apical dendrites), D: Subtree C (soma and basal dendrites). The effect of the EPSP pairing is apparent in the distal dendrites (subtree C, second column), where the inferred amplitude is considerably higher that in the plain 10 bAP protocol.

The first row (A) of Fig. 6 shows the detailed trace of the normalized measurements and inferred normalized concentrations for one point in subtree B (marked with a blue electrode in Fig. 6C), for the full length of the experiment. Panels B,C and D show the normalized measurements and inferred traces at one location of the subtrees A,B and C respectively for the first 2 seconds of the experiment. These points are highlighted with a blue electrode in the second and fourth columns. The 1st column is for the “10 bAP protocol” and the 3rd for the “bAP+EPSP protocol”. The red dashed lines mark the timing of the stimulation that invoked the action potentials. Apart from the significant denoising, the algorithm predicts the timing of the bAPs and provides an estimate for the amplitude of the transient due to the bAP, relative to the baseline concentration at this location.

In some cases, there are also a few spikes detected outside the 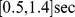 excitation window. The main reason for this is the data is non stationary. The baseline concentration is different before and after the excitation, with the latter being usually higher. This can also be seen in Fig. 6A that shows the measured and inferred traces over the whole course of the experiment. However, in our algorithm, the baseline activity is kept constant, and is estimated as the mean measured value at the first 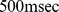. As a result, after the end of the excitation window the inferred trace tends to relax to a lower value than the one suggested by the data. This is prevented by the inferred spikes outside the excitation window. Note that sometimes these spikes, although not experimentally caused, are also suggested by the data. However, these spikes have always small amplitude and are spatially isolated in the sense that they are not inferred at multiple locations in the tree at similar times (as can be seen in the movies online). Consequently, they can be easily classified as “artificial” during post-processing of the data.

A possible solution to prevent such spikes would be to set the spike rate outside the excitation window to a very low value. However this led to overfitting in the amplitude of the spikes in the interval 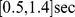. More specifically, if outside spikes are prohibited then the last spike at 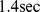 will be overestimated, so that the inferred trace did not relax to the actual baseline value of the model until the end of the experiment. Our method allows (possibly spurious) spikes at other times as well, but is robust to the length of the experiment in the sense that the amplitude of the transients remained approximately constant, independent of the length of the imaging data that was analyzed. Another possible approach would be to model the baseline as a slow time varying hidden variable and infer it together with the vector of the hidden states 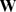. However, this would significantly complicate our model, since the 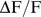 transformation would no longer be readily applicable.

From Fig. 6 we also see that the amplitude of the last transient in the bAP+EPSP protocol is larger than the corresponding one for the bAP protocol, especially in the locations away from the soma in the tuft (Fig. 6B). This is further investigated in the second and fourth columns where the amplitude of the last transient is plotted in the whole subtree for all subtrees and both experimental protocols. This amplitude was computed by subtracting the inferred calcium signal just before the last spike from the inferred calcium signal 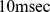 after the last spike. From these two columns of Fig. 6 we see that for the subtrees B and C, the amplitude of the last transient is larger in the bAP+EPSP protocol than the control case (bAP only). This supports the hypothesis that calcium plays an important role in the interplay between backpropagating action potentials (bAPs) and excitatory post-synaptic potentials (EPSPs) that mediate synaptic strength changes.

**Figure 7:**
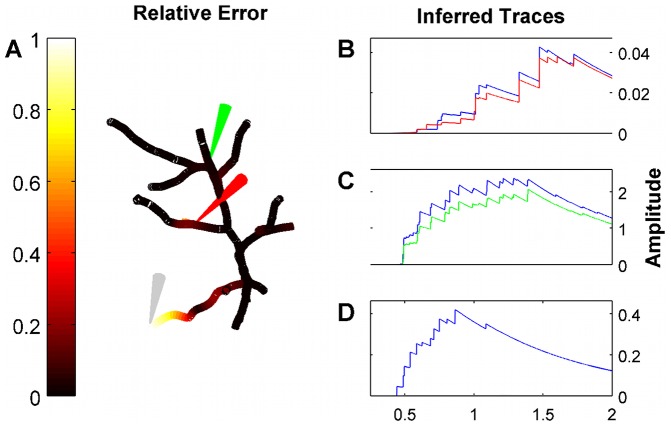
Cross-validation by leaving out locations. A: Locations of the omitted imaged sites and relative error between “full” and “cross-validated” results along the tree. B–D: Comparison of the “full” traces (blue) with the cross-validated traces at the omitted locations. The colors of the “cross-validated” traces are in correspondence with the colors of the fictional electrodes in panel A. The algorithm can predict the traces at left out imaging sites that are located in the middle of dendritic branches by interpolating the results from neighboring measured locations. However the traces of omitted compartments located at the end of branches cannot be inferred.

As an additional test of the robustness of our results we examined how removing measurements from selected locations affects the inferred traces. In Fig. 7 we show the results for subtree A under the bAP protocol, when omitting data from 3 measurement locations. These locations are indicated with the colored electrodes in Fig. 7A and the inferred traces at these locations are plotted with the same colors in Fig. 7B–D respectively. For comparison purposes we also plot with blue the inferred traces when all the data was considered from the algorithm.

The results shown in Fig. 7 are intuitive: When the measurement sites that are left out are located at points that fall between locations from which measurements are available (red and green electrodes, Fig. 7B–C), the algorithm can approximate the bound probe molecule traces. The prior we used causes the newly inferred traces to have generally smaller amplitude. The event times are again inferred and are in good alignment with the event times inferred when all of the data is used. However, when we try to infer traces at the endpoints of dendritic branches (Fig. 7D) and/or at locations far from those at which measurements are available, the inferred traces are shrunk to zero by the prior, since there is no data available to encourage non-zero estimates in this case. Finally in Fig. 7A we also plot the relative error between the “full” and “cross-validated” traces for all the compartments of the subtree. The relative error for location x is defined as 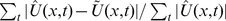, where 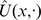 denotes the inferred trace at location 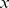 when all the data is considered and 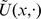 is the inferred trace when the chosen locations have been left out. As expected from the spatially localized effect of any measurement to our algorithm, the inferred traces at compartments that are far from all the left out locations do not change. As a technical side note, we point out that when trying our algorithm for this cross-validation experiment, we did not reestimate all the necessary parameters as a means to facilitate direct comparison. Estimating these parameters again could lead to a better suited choice of spline basis 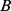 and potentially to better interpolation results.

**Figure 8:**
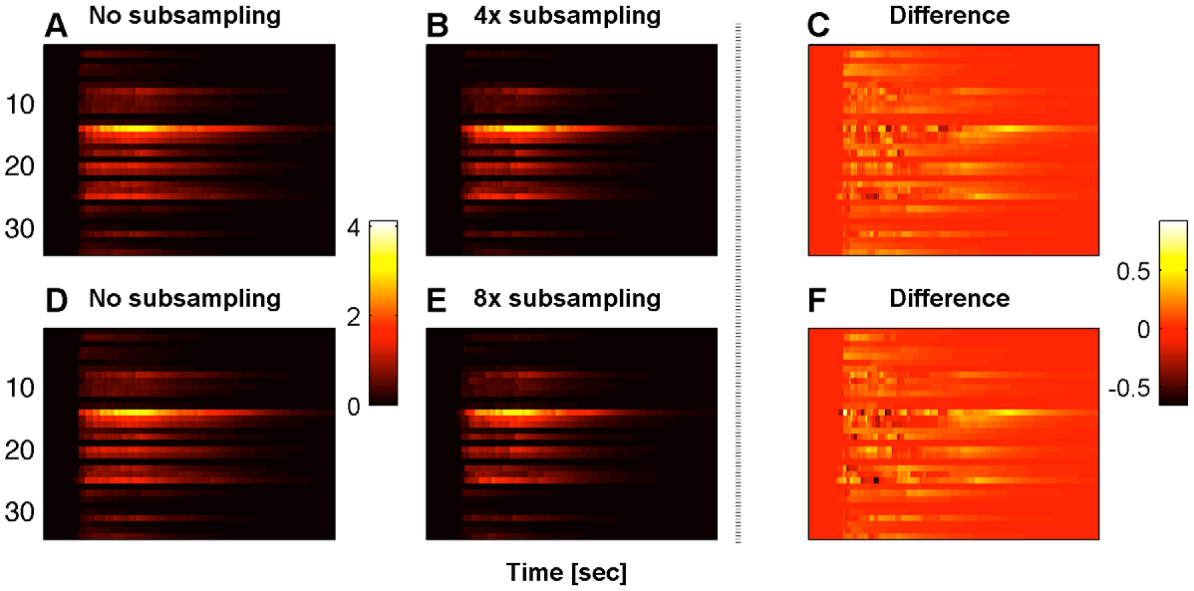
Effect of the temporal sampling rate. A and D: Inferred traces using all the data for subtree B under the bAP protocol. B and E: Inferred traces after 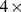 and 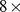 subsampling, respectively. C and F: Differences of the inferred traces under subsampling conditions from the traces that are inferred using all the data. Note that many qualitative features of the inferred probe molecule profile are preserved even given heavy subsampling.

Finally, we examined the effect of the temporal sampling rate, by applying our algorithm to a subsampled version of the data. In Fig. 8 we examine the differences in the results when the data have been subsampled 4 and 8 times (subtree B under the bAP protocol). As expected, the difference between the full and subsampled results increase with subsampling factor. Using subsampled data, the event times are not always predicted. However, as can be seen from Fig. 8, the general behavior of the inferred traces remains largely the same. It is also important to note that in an experimental situation a lower temporal sampling rate typically leads to a higher signal to noise ratio (SNR). It would be interesting examine this quality-quantity tradeoff more thoroughly [5]; however, such an analysis is not directly possible with this dataset, since simple subsampling does not change the SNR. The analysis shown in Figs. 7 and 8 was also carried out for the other subtrees and experimental protocols and no qualitative differences were observed (data not shown).

### Application to simulated data under “synaptic bombardment” conditions

**Figure 9:**
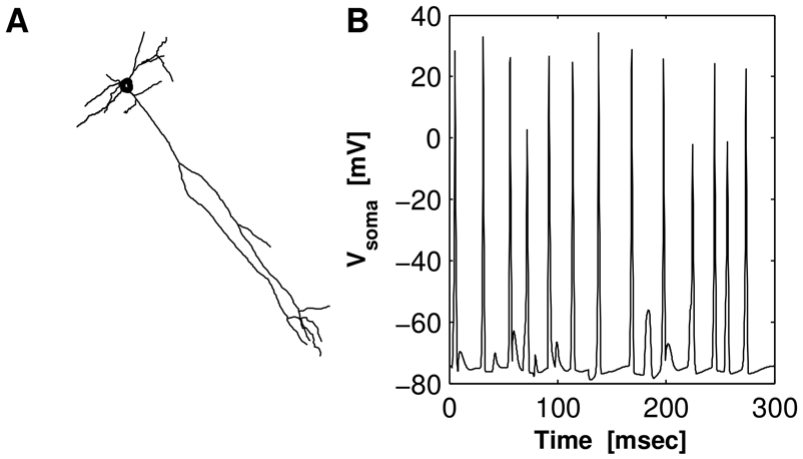
Morphology of the CA1 neuron and somatic voltage trace used in **Fig. 10**. A: Morphology of the CA1 neuron in the x-y axis. B: Voltage trace at the soma. 80 synaptic events were simulated and the neuron produced 10 action potentials. Neuron model and code adapted from [30].

We finally applied our algorithm to simulated data in a dendritic tree receiving multiple synaptic input from both excitatory and inhibitory presynaptic neurons. (These simulations are necessarily somewhat underconstrained, since the spatiotemporal structure and effect of in-vivo inputs is only beginning to be characterized [28],[29].) We simulated data from a rat CA1 reconstructed tree model (Fig. 9; 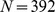 compartments) used in [30] (ch. 9), where the dendrites were endowed with the standard Hodgkin Huxley neuron channels [31] and the voltage profile obeyed the active dendritic cable equations [32]. To this model we also added T-type voltage dependent calcium channels with the same parameters as the ones measured experimentally in [33]. (Clearly more elaborate and realistic models are possible, but as we will see below the details of the voltage and calcium model seemed to have a minimal effect on the inference quality here.) We placed a total number of 40 synapses (both excitatory and inhibitory) at random locations along the tree. The synapses were of alpha type with 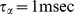. The synapses were activated at random times and a total of 80 synaptic events were produced. The neuron was simulated for 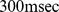, during which 10 action potentials were triggered as a result of the synaptic inputs; each of these spikes propagated back into the tree to a varying extent.

**Figure 10:**
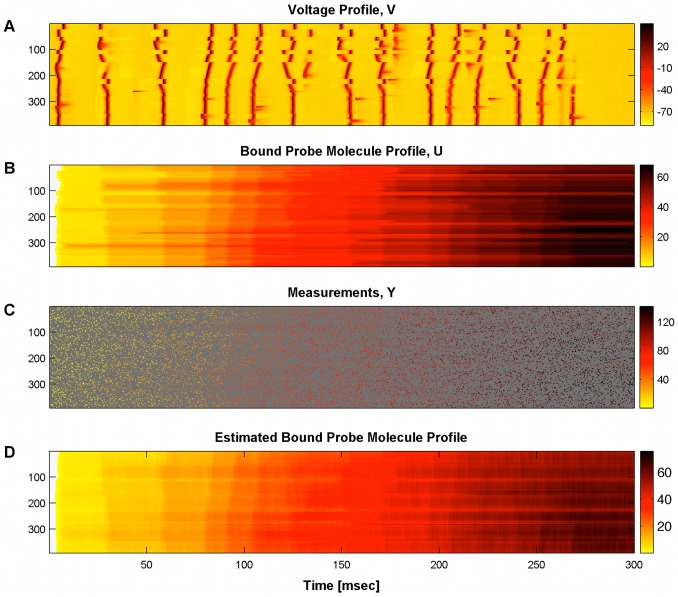
Calcium estimation under “synaptic bombardment” conditions. A: Spatiotemporal voltage profile. B: Bound probe molecule profile: C: Measurement locations and values at each timestep (dark grey corresponds to locations that were not imaged at each timestep). D: Estimated bound probe molecule profile. The algorithm estimates satisfactorily the general behavior of the bound probe molecule concentration profile.

Every 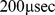 we obtained ten noisy measurements from uniformly random locations, using the same measurement noise distribution described above (6). The probe molecule baseline concentration and the time constant of the dye were both assumed known. To apply our algorithm we constructed a spatial smoothing basis consisting of 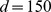 spline functions. For each of the hidden “spike” variables 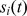 we used a simple uncorrelated exponential prior at each location with a rate equal to the firing rate of the neuron. The results of the inference are shown in Fig. 10: even with a simple uncorrelated prior for 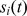, and no detailed knowledge of the underlying biophysical voltage and calcium dynamics, the algorithm does a satisfactory job in estimating the bound probe molecule concentration profile.

## Discussion

We presented a statistical model for spatiotemporal calcium estimation (up to an affine transformation) given single-trial, spatially localized, noisy imaging measurements. We showed that our algorithm can infer the timing of bAPs and provide estimates about the amplitude of the transients that they initiate. Although the optical measurements come from a small subset of the neuron's compartments the algorithm estimates the full spatiotemporal calcium profile. Moreover, it runs with complexity that scales linearly both with the length of the experiment and the size of the tree, and as a result it can be used in arbitrarily long experiments and large dendritic trees. Our full spatiotemporal smoothing algorithm therefore enables the study of selective propagation of bAPs into specific dendrites, and of the effects of calcium as a coincidence indicator between bAPs and EPSPs. Although the focus of this study was the study of the interactions between bAPs and known EPSP stimulation, we also tested the generality of our algorithm by applying it to a conductance based neuron driven by random (unobserved) synaptic inputs. More generally, our algorithm opens up the possibility of single-trial analysis of spatiotemporal calcium dynamics imaged on large dendritic trees, and could therefore potentially lead to a better understanding of these complex dynamics.

To obtain a relatively simple model and an efficient inference algorithm, we had to make a number of assumptions. For example, we employed a functional approach by assuming that the relative bound probe molecule concentration can be spatially approximated by a sum of smooth spline functions on the tree. This is reasonable since calcium diffuses locally through the dendritic tree. We assumed that the baseline fluorescence is constant before and after the spike stimulation. While this is true in general, we observed that in some locations (and especially the ones in the main dendritic trunk), the baseline can change significantly after the excitation, leading to the inferred spikes outside the excitation window, as discussed in the Results. The model also relies on the assumption that the bound probe molecules do not saturate during repetitive excitation, and that the rate of calcium unbinding remains constant. Analysis shows that the amplitude of the transients in general decreases as more spikes are applied. This has been reported in many studies (e.g. [34]). However, in some situations (see for example the first row in Fig. 6b) there are indications of fast relaxations after the last spike which may imply saturating effects.

Apart from constructing a spatial basis for the relative bound probe molecules, we also tried the same approach on the un-normalized, raw bound probe molecule measurements. However, we noticed that in this case the required dimension 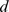 of the spline basis was much larger because of the large variations in the observed measurements. Consequently, the resulting spline functions were very localized and as a result, inference suffered from overfitting. Moreover, the noise statistics varied considerably at the different measurement locations. Many bAP induced transients went undetected, especially at the distal dendrites, because of their low amplitude. As a result, we concluded that normalizing the measurements with the estimated baseline fluorescence concentration greatly improved the quality and interpretability of results. This approach also has a clear interpretation since the normalized measurements are linearly related to the underlying calcium concentration.

Our model, so far, assumes only temporal dependencies on the hidden activation states 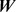. Allowing spatial dependencies in 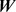 enables the modeling of calcium propagation along the dendritic tree (similar to the cable equation for voltage diffusion) and would lead towards a more biophysical realistic model. To allow this we could modify the state equation Eq. (15) as
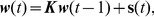
(27)where 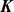 is a suitable stable matrix. In the simplest case the spatial dependencies are restricted to the direct neighbors of each hidden variable, as in the standard cable equation [35]. In this case 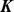 retains the tree structure. Our fast algorithm can be applied in this setup without losing its linear 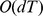 complexity, using methods similar to the ones developed in [36] for voltage measurements in dendritic trees.

The prior on the transient amplitude used in our model was very simple. It reflected only the knowledge of the interval over which the bAPs were initiated and their rate, but not the exact timing of the bAPs and other qualitative characteristics of the evoked transients. A more sophisticated prior can be chosen that exploits several characteristics of the bAPs. For example, the exact knowledge of the bAP timings can be incorporated to a greater extent by manipulating the time varying spike rate so that it becomes high in the time windows when we expect spikes, and low elsewhere. Moreover, at the dendrites, the amplitude of spikes usually decreases with the distance from the soma. In our approach “we let the data speak”, i.e., we determine the prior of the spike amplitude from the recordings as we discussed in the Results section. However, we can further impose a decreasing amplitude constraint as a regularizer to further promote this behavior, to better deal with the case where individual dendrites are very sparsely imaged. In matrix-vector notation this can be written as 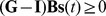 for all 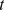, where 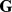 is the directional adjacency matrix of the neuron graph [24]. The directional adjacency matrix is defined as 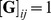 if the compartment 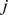 is the parent of compartment 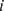 and 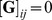 otherwise. Since 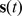 represents the bumps of the hidden states at time 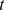, 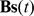 represents the bumps on the bound probe molecule concentration. As the root (soma) of the tree has no parents, the first row of the vector 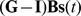 does not correspond to a difference between amplitudes and must be omitted from the constraints. Alternatively, we can set 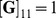 to make it trivial. The matrix 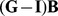 has a tree-stucture and such a constraint does not affect the complexity of our algorithm. Additionally, the exponential prior distribution that we used promotes sparsity among the bumps. By introducing a group-sparse nonnegative prior [37] we could promote synchronized bumps along the tree, again without changing the linear complexity per iteration [38]. However, note that although our algorithm does not promote the above qualitative characteristics, these are observed in the inferred traces, indicating its suitability in cases with limited prior information.

One natural question is whether the full spatiotemporal profile of calcium concentration can also be estimated. Our model promotes the inference of sparse events; this sparse regularization is necessary to avoid overfitting artifacts given our undersampled measurements. These sparse events, in turn, are interpreted as indicators of calcium concentration transients which are assumed to be instantaneous. As a result, due to these simplifications the detailed calcium dynamics cannot easily be inferred from our algorithm at its current form; see the Methods section for further discussion. Additional constraints on the model (e.g., a modification of the prior based on some knowledge of synaptic input locations) could improve this estimation, but this remains a direction for future work.

A final interesting question concerns the design of an optimal path for measuring at different locations across the tree. In our dataset the set of imaged sites was chosen by the experimenter and held fixed for each imaging session. Such an approach is suboptimal since it leaves many sites unrecorded and also neglects the spatial correlations along the tree. A heuristic method was presented in [39] for the case of imaging a population of neurons. The goal there was to minimize the length of the scanning path in order to provide faster measurements. In our case of single neuron imaging, where the path length is small and fast measurements can be obtained through RAMP microscopy, the optimality criterion should be the one of maximally informative measurement locations. A similar method was presented in [40] for the case of voltage filtering. However the extension of this method to calcium measurements is challenging because of our non-Gaussian setup and the added nonnegativity constraints. The directions mentioned above will be pursued in future work.

## Supporting Information

Text S1 Description of the log-barrier method for the fast solution of our MAP estimation problem. Discussion of the banded structure of the Hessian and how to exploit it for the fast computation of the Newton direction.
(PDF)
