# Supplementary material for: Fast Spatiotemporal Smoothing of Calcium Measurements in Dendritic Trees
Source: PLoS Comput Biol. 2012 Jun 28;8(6):e1002569. doi: 10.1371/journal.pcbi.1002569 (PMC3386185; doi:10.1371/journal.pcbi.1002569)
Supplement: Text S1 — Description of the log-barrier method for the fast solution of our MAP estimation problem. Discussion of the banded structure of the Hessian and how to exploit it for the fast computation of the Newton direction. (PDF) [file pcbi.1002569.s001.pdf]

Supporting Information for: Fast Spatiotemporal Smoothing of Calcium Measurements in Dendritic Trees

Eftychios A. Pnevmatikakis[1*], Keith Kelleher[2†], Rebecca Chen[2], Peter Saggau[3], Krešimir Josić[2], Liam Paninski[1]
1 Department of Statistics and Center for Theoretical Neuroscience, Columbia University, New York, NY, USA
2 Department of Mathematics, University of Houston, Houston, TX, USA
3 Department of Neuroscience, Baylor College of Medicine, Houston, TX, USA
∗ E-mail: eftychios@stat.columbia.edu
† Current address: Salk Institute for Biological Studies, La Jolla, CA, USA

Supporting information

MAP estimation with a log-barrier method

To find the MAP estimate we need to solve the following optimization problem

$$\hat{\boldsymbol{W}}_{\text{MAP}} = \underset{\boldsymbol{W}:\boldsymbol{w}(1)\geq\boldsymbol{0},\boldsymbol{w}(t)-\gamma\boldsymbol{w}(t-1)\geq\boldsymbol{0}}{\arg\max} \left\{\log p(\boldsymbol{Y}|\boldsymbol{W}) + \log p(\boldsymbol{W})\right\}. \tag{1}$$

The vector of differences $[\boldsymbol{w}(2)-\gamma\boldsymbol{w}(1);\ldots;\boldsymbol{w}(T)-\gamma\boldsymbol{w}(T-1)]$ can by written for convenience as a matrix-vector multiplication $\boldsymbol{DW}$, where the $d(T-1)\times dT$ matrix $\boldsymbol{D}$ is defined as $\boldsymbol{D}=\boldsymbol{D}_l\otimes I_d$ and $\boldsymbol{D}_l$ is a $(T-1)\times T$ matrix with

$$[\boldsymbol{D}_l]_{ij} = -\gamma\delta_{ij} + \delta_{i+1,j},$$

where $\otimes$ denotes the tensor product, I_d is an identity matrix with dimension d, and δ_{kl} the Kronecker delta, i.e., $\delta_{kl}=1$ if $k=l$ and 0 otherwise. More explicitly, $\boldsymbol{D}$ is a $d\times d$-block diagonal matrix, where each block (in the diagonal) is equal to $\boldsymbol{D}_l$.

To enforce the inequality constraints we can use a log-barrier method [1] similar to that discussed in [2]. Briefly, the idea behind a barrier method is as follows. The hard nonnegativity constraints are introduced in the log-posterior function using a barrier function (in this case the logarithm) that becomes infinitely negative when these constraints are violated. This term is also weighted by a positive scalar z.

$$\hat{\boldsymbol{W}}_z = \underset{\boldsymbol{W}}{\arg\max}\left\{L_z(\boldsymbol{W})\right\},\ \text{with } L_z(\boldsymbol{W}) = \log p(\boldsymbol{W}|\boldsymbol{Y}) + z\left(\boldsymbol{1}_{d(T-1)}^T\log(\boldsymbol{DW}) + \boldsymbol{1}_d^T\log(\boldsymbol{w}(1))\right) \tag{2}$$

Note that the logarithms in the barrier term on the right side of Eq. (2) are taken element-wise and $\boldsymbol{1}_d, \boldsymbol{1}_{d(T-1)}$ denote vectors of ones of dimensions given by the subscripts. Now for the given z we solve the optimization problem and obtain a solution $\hat{\boldsymbol{W}}_z$. We then decrease z by dividing it with a constant factor (e.g. 10) and solve again Eq. (2) for the new value of z using warm starts, i.e., using the previously computed solution as the initial point. Each of these computed solutions lies on the interior of the feasible set, i.e., all the nonnegativity constraints are satisfied with strict inequalities. The theory of interior point methods guarantees that as z approaches to 0, the solution converges fast to the true MAP estimate [3]:

$$\hat{\boldsymbol{W}}_{\text{MAP}} = \lim_{z\to 0^+}\hat{\boldsymbol{W}}_z. \tag{3}$$

Eq. (2) is strictly concave and twice differentiable and therefore can be efficiently maximized using Newton's method. Intuitively, at the $(n+1)$-th iteration, Newton's method approximates $L_z(\boldsymbol{W}_n)$ as a quadratic function around the point $\boldsymbol{W}_n$ and then updates the estimate by moving $\boldsymbol{W}_n$ along the direction that minimizes the quadratic approximation:

$$\boldsymbol{W}_{n+1} = \boldsymbol{W}_n - t_n H^{-1}\nabla, \tag{4}$$

where H, ∇ denote the value of the Hessian and gradient of $L_z(\boldsymbol{W})$ evaluated at the point $\boldsymbol{W}_n$ and the stepsize $t_n \in [0,1]$ is chosen to ensure that the objective increases on each iteration. The gradient and the Hessian (computed at the point $\boldsymbol{W}$) are given by

$$\nabla = \frac{\partial}{\partial \boldsymbol{W}} L_z(\boldsymbol{W}) = \frac{\partial}{\partial \boldsymbol{W}} \log p(\boldsymbol{Y}|\boldsymbol{W}) + \frac{\partial}{\partial \boldsymbol{W}} \log p(\boldsymbol{W}) + z\left(\boldsymbol{D}^T (\boldsymbol{D}\boldsymbol{W}).^{-1} + \boldsymbol{w}(1).^{-1}\right)$$
$$H = \frac{\partial^2}{\partial \boldsymbol{W}^2} L_z(\boldsymbol{W}) = \frac{\partial^2}{\partial \boldsymbol{W}^2} \log p(\boldsymbol{Y}|\boldsymbol{W}) + \frac{\partial^2}{\partial \boldsymbol{W}^2} \log p(\boldsymbol{W}) - z\left(\boldsymbol{D}^T \mathrm{diag}\{(\boldsymbol{D}\boldsymbol{W}).^{-2}\}\boldsymbol{D} + \mathrm{diag}\{\boldsymbol{w}(1).^{-2}\}\right). \tag{5}$$

The Newton method requires only a few iterations to converge to the maximizer [1]. However, the computation of the Newton direction $H^{-1}\nabla$ at each iteration is computationally expensive step, since it requires a matrix solve of size $dT \times dT$, where d is the number of spline functions used. Because of the state-space structure of the model, the Newton direction can be computed with cost $O(d^3T)$ in time and $O(d^2T)$ memory requirement, using standard forward-backward schemes as discussed in [4]. By exploiting the tree-banded structure of $\mathbf{B}$ and the spatially and temporally localized nature of the measurements, we can reduce the complexity to $O(dT)$, which allows us to apply our algorithm in trees of arbitrary size. We next discuss the derivation of this approach.

Exploiting bandedness for computation of the Newton direction

As noted above, the most expensive step of our algorithm is the determination of the Newton direction via $H^{-1}\nabla$ (see Eq. (4)). The Hessian is symmetric and negative definite (since the log-posterior is strictly concave). To examine the structure of the Hessian we look at the three terms in Eq. (5) separately. Since the observations at each time t are instantaneous imaging measurements, they depend only on the state variable at that time t. Thus, the first term of Eq. (5) contributes a block diagonal matrix to the Hessian. The second term of the sum, because of the Markovian structure, contributes a block-tridiagonal matrix to the Hessian [4]. Moreover, if we assume spatial independence between the activation parameters $w_i(t)$, then each block of the tridiagonal-matrix is a diagonal matrix itself. For the third term of the Hessian let $\mathrm{spy}(A)$ denote the set of nonzero entries of a matrix A, i.e.,

$$\mathrm{spy}(A) = \{(i,j) : [A]_{ij} \neq 0\}. \tag{6}$$

Then

$$\mathrm{spy}(\boldsymbol{D}^T \mathrm{diag}\{(\boldsymbol{D}\boldsymbol{W}).^{-2}\}\boldsymbol{D} + \mathrm{diag}\{\boldsymbol{w}(1).^{-2}\}) \subseteq \mathrm{spy}(\boldsymbol{D}^T\boldsymbol{D}) = \mathrm{spy}((\boldsymbol{D}_l^T\boldsymbol{D}_l) \otimes I_d), \tag{7}$$

where $\otimes$ denotes the tensor product and I_d is an identity matrix of dimension d. From (7) we see that the sparsity profile of the third term does not depend on the state variable $\boldsymbol{W}$, but only on the Markovian structure of our state-space model. Moreover,

$$\begin{aligned} \mathrm{spy}(\boldsymbol{D}_l) &= \{(i,i),(i,i+1), i = 1,\dots,T-1\} \Rightarrow \\ \mathrm{spy}(\boldsymbol{D}_l^T\boldsymbol{D}_l) &= \{(i,j) : |i-j| \leq 1, 1 \leq i,j \leq T\} \Rightarrow \\ \mathrm{spy}(\boldsymbol{D}^T\boldsymbol{D}) &= \{(i,j) : i-j = -d, 0, d, 1 \leq i,j \leq dT\}. \end{aligned} \tag{8}$$

Therefore this term of the Hessian contributes a block-tridiagonal matrix where each nonzero block is a diagonal matrix. Thus we see that the Hessian is in general a block tridiagonal matrix:

$$H = \begin{bmatrix} H_1 & Q_1 & 0 & \dots & 0 \\ Q_1^T & H_2 & Q_2 & \dots & 0 \\ & \ddots & \ddots & \ddots & \\ 0 & 0 & \dots & H_{T-1} & Q_{T-1} \\ 0 & 0 & \dots & Q_{T-1}^T & H_T \end{bmatrix}, \tag{9}$$

Such matrices can be efficiently inverted (using e.g. LDL^T decomposition [5]) in $O(d^3T)$ time and space. In general, we may write $H = LDL^T$, with

$$L = \begin{bmatrix} I & 0 & \dots & 0 & 0 \\ L_2 & I & \dots & 0 & 0 \\ 0 & \ddots & \ddots & \ddots & 0 \\ 0 & 0 & \dots & L_T & I \end{bmatrix}, \quad D = \begin{bmatrix} D_1 & 0 & \dots & 0 \\ 0 & D_2 & \dots & 0 \\ 0 & \ddots & \ddots & 0 \\ 0 & 0 & \dots & D_T \end{bmatrix}. \tag{10}$$

These matrices satisfy the system of equations [5]:

$$\begin{aligned} D_1 &= H_1 \\ D_{t-1}L_t^T &= Q_{t-1}, \;\; t = 2, \dots, T \\ L_t D_{t-1} L_t^T + D_t &= H_t, \;\; t = 2, \dots, T. \end{aligned} \tag{11}$$

By denoting as $A \backslash b$ a solution of a system of linear equations $Ax = b$ with respect to x, the Newton direction $\boldsymbol{x} = H \backslash \nabla$ can be computed using the forward-backward recursion:

$$\begin{aligned} \boldsymbol{y}(1) &= \nabla(1) \\ \boldsymbol{y}(t) &= \nabla(t) - L_t \boldsymbol{y}(t-1), \quad t = 2, \dots, T \\ \boldsymbol{x}(T) &= D_T \backslash \boldsymbol{y}(T) \\ \boldsymbol{x}(t) &= D_t \backslash \boldsymbol{y}(t) - L_{t+1}^T \boldsymbol{x}(t+1), \quad t = T-1, \dots, 1. \end{aligned} \tag{12}$$

The computational complexity of the forward backward scheme scales linearly with time. The matrices that we need to solve are the negative definite matrices D_t; therefore, in the worst case scenario, the complexity of the algorithm is $O(d^3T)$ in computational time and $O(d^2T)$ in space requirement since the matrices D_t, L_t need to be stored.

In our case this complexity can be further reduced by exploiting the tree structure and the fact that the measurements are also spatially localized. Each block H_t on the diagonal of the Hessian is equal to a diagonal matrix plus a component due to the measurements, equal to

$$\frac{\partial^2}{\partial \boldsymbol{w}(t)^2} \log p(\boldsymbol{y}(t)|\boldsymbol{w}(t)) = \boldsymbol{B}_t^T \boldsymbol{E}_t^{-1} \boldsymbol{B}_t, \tag{13}$$

where $\boldsymbol{B}_t$ is the measurement matrix at time t, and $\boldsymbol{E}_t$ is the measurement noise covariance matrix at time t, which is diagonal since the measurements are assumed to be independent.

As described above, the matrix $\boldsymbol{B}$ is constructed by the spatial discretization of the spline functions f_i. Since the functions f_i have limited spatial support, every column of $\boldsymbol{B}$, and thus the matrix $\boldsymbol{B}$ itself, is sparse. In the simplest case where the cell consists of a single unbranched dendritic segment, $\boldsymbol{B}$ is a banded matrix which allows for fast matrix vector multiplications and inversions [6]. In general, we are looking at a full dendritic tree, and $\boldsymbol{B}$ is *tree-banded*, in the sense that $\boldsymbol{B}_{ic(j)} = 0$ if the i-th compartment is sufficiently distant along the tree from the j-th bump center $c(j)$. Note that similarly to banded matrices, fast computations can also be performed with tree-banded matrices. For example, matrix solvers can run in linear time by performing Gaussian elimination on individual branches starting from the leaves and ending in the root [7]. Now each $\boldsymbol{B}_t$ is formed by choosing and scaling the rows of $\boldsymbol{B}$ that corresponds to the compartments measured at each time, and thus remains tree-banded as well. From Eqs. (8) and Eq. (13) it follows that each diagonal block of the Hessian H_t will also be tree-banded.

Apart from being tree-banded, the diagonal blocks H_t of the Hessian have also most of their energy concentrated along their main diagonal. This is due to three reasons: First, the spline function attains its maximum around its bump center, i.e., we expect $[\boldsymbol{B}]_{ic(i)} > [\boldsymbol{B}]_{jc(i)}$ for $i \neq j$, where $c(i)$ denotes

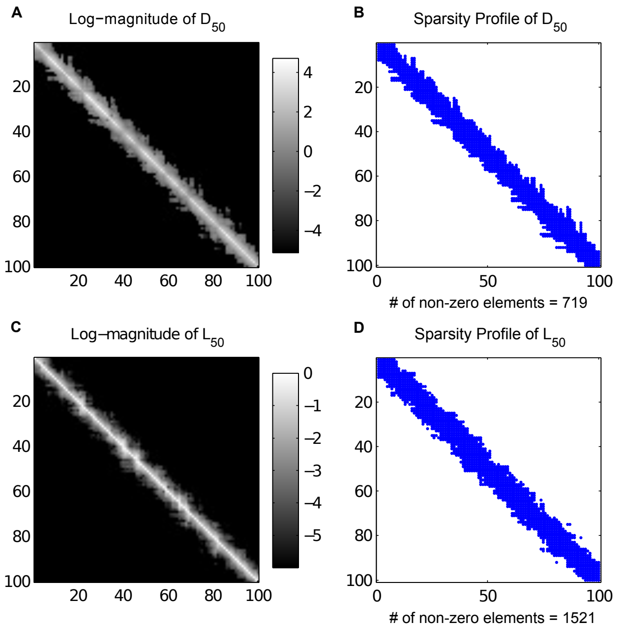

Figure 1. Sparsity profiles of the LDL^T decomposition at timestep $t = 50$. Logarithm of the magnitude of the entries of matrices $\boldsymbol{D}_{50}$ (A) and $\boldsymbol{L}_{50}$ (C). The magnitude is plotted in a logarithmic scale to demonstrate its rapid decrease away from the main diagonal. Sparsity profile and number of non-zero elements for $\boldsymbol{D}_{50}$ (B) and $\boldsymbol{L}_{50}$ (D), after the adaptive thresholding. The components remain approximately banded with most of their energy concentrated along the main diagonal.

the center of the i-th spline function. As a result, and since the measurements at each time step are independent, the matrices $\boldsymbol{B}_t^T \boldsymbol{E}_t^{-1} \boldsymbol{B}_t$ of (13) will have most of their energy along the main diagonal. Second, the prior on each $\boldsymbol{w}(t)$ is spatially uncorrelated and therefore $\partial^2 \log p(\boldsymbol{w}(t))/\partial \boldsymbol{w}(t)^2$ is diagonal. Third, the barrier term acts only on the temporal differences $\boldsymbol{w}(t) - \gamma \boldsymbol{w}(t-1)$ and therefore has an effect only on the main diagonal of the Hessian (see Eq. (8)). Now since the matrices H_t have most of their energy concentrated along their main diagonal, apart from being tree-banded, it follows that their inverses will also remain approximately banded with most of their energy concentrated on their main diagonal. To see that consider the simple case of a tridiagonal matrix that is diagonally dominant. In this case, its inverse has most of its energy in its main diagonal, and the magnitude of the entries falls exponentially with their distance from the main diagonal. As a result it can be well approximated with a banded matrix. Our case is a straightforward generalization of this result.

Now, with H_t and H_{t-1} (approximately) tree-banded, and the off diagonal blocks of the Hessian Q_t diagonal (see Eq. (8)), it is easy to see that the matrices L_t, D_t in the LDL^T recursion Eq. (11) can also be approximated by tree-banded and banded matrices respectively. Consequently, all the matrices in the forward-backward scheme remain approximately tree banded (with effective bandwidth that does not depend on d) and therefore the computation of the Newton direction using the forward-backward scheme of Eq. (12) can be practically computed in $O(dT)$ time. For similar reasons, the storage requirements of the algorithm also scale linearly with d and T. Note that, as discussed above, we can relax the spatial independence assumption on each $\boldsymbol{w}(t)$; imposing local spatial correlations in $\boldsymbol{w}(t)$ would again lead to a tree-banded structure, albeit with slightly larger matrix bandwidth.

We illustrate the banded property of the LDL^T decomposition in a simple setup where we are looking at a single linear dendrite with $d = 100$ and $T = 100$. In this case, $\boldsymbol{B}$ and the diagonal blocks of the

Hessian are banded matrices. Fig. 1 shows the magnitude (in a logarithmic scale) and sparsity profiles of D_{50} and L_{50}, from the LDL^T decomposition recursion Eq. (11). As can be seen the energy of the matrices is mostly concentrated on the main diagonal and therefore the matrices remain approximately banded. Note that the matrices are only approximately banded and some thresholding is required to make them sparse. In the case of the diagonal matrices D_t we perform adaptive thresholding on the Cholesky factor of $-D_t$ (each D_i is negative definite), via the cholinc function in Matlab. The matrices L_t are thresholded directly, since this direct thresholding of L_t will not impact the symmetry or negative definiteness of LDL^T, by construction.

References

1. Boyd S, Vandenberghe L (2004) Convex Optimization. Oxford University Press.

2. Vogelstein J, Packer A, Machado T, Sippy T, Babadi B, et al. (2010) Fast non-negative deconvolution for spike train inference from population calcium imaging. Journal of Neurophysiology 104: 3691-3704.

3. Nesterov Y, Nemirovskii A (1987) Interior-point polynomial algorithms in convex programming, volume 13. Society for Industrial Mathematics.

4. Paninski L, Ahmadian Y, Ferreira D, Koyama S, Rahnama K, et al. (2010) A new look at state-space models for neural data. Journal of Computational Neuroscience 29: 107-126.

5. Brockwell P, Davis R (1991) Time Series: Theory and Methods. Springer.

6. Golub G, Van Loan C (1996) Matrix computations. Johns Hopkins Univ Press.

7. Hines M (1984) Efficient computation of branched nerve equations. International Journal of Bio-Medical Computing 15: 69 - 76.
